# Isoniazid resistance levels of *Mycobacterium tuberculosis* can largely be predicted by high-confidence resistance-conferring mutations

**DOI:** 10.1038/s41598-018-21378-x

**Published:** 2018-02-19

**Authors:** Pauline Lempens, Conor J. Meehan, Koen Vandelannoote, Kristina Fissette, Pim de Rijk, Armand Van Deun, Leen Rigouts, Bouke C. de Jong

**Affiliations:** 1 Unit of Mycobacteriology, Department of Biomedical Sciences, Institute of Tropical Medicine, Antwerp, Belgium; 2 Department of Biomedical Sciences, University of Antwerp, Antwerp, Belgium

## Abstract

The majority of *Mycobacterium tuberculosis* isolates resistant to isoniazid harbour a mutation in *katG*. Since these mutations cause a wide range of minimum inhibitory concentrations (MICs), largely below the serum level reached with higher dosing (15 mg/L upon 15–20 mg/kg), the drug might still remain partly active in presence of a *katG* mutation. We therefore investigated which genetic mutations predict the level of phenotypic isoniazid resistance in clinical *M. tuberculosis* isolates. To this end, the association between known and unknown isoniazid resistance-conferring mutations in whole genome sequences, and the isoniazid MICs of 176 isolates was examined. We found mostly moderate-level resistance characterized by a mode of 6.4 mg/L for the very common *katG* Ser315Thr mutation, and always very high MICs (≥19.2 mg/L) for the combination of *katG* Ser315Thr and *inhA* c-15t. Contrary to common belief, isolates harbouring *inhA* c-15t alone, partly also showed moderate-level resistance, particularly when combined with *inhA* Ser94Ala. No overt association between low-confidence or unknown mutations, except in *katG*, and isoniazid resistance (level) was found. Except for the rare *katG* deletion, line probe assay is thus not sufficiently accurate to predict the level of isoniazid resistance for a single mutation in *katG* or *inhA*.

## Introduction

Effective control of tuberculosis (TB) is especially complicated among patients with multidrug-resistant TB (MDR-TB), which is characterized by resistance to at least isoniazid and rifampicin, the two most powerful drugs against TB, used in standard first-line treatment^1,2^. Preventing the activation of the pro-drug isoniazid, mutations in *katG* are the most frequent cause of isoniazid resistance^3,4^. Although more than 300 different *katG* mutations have been identified, mutations at codon 315 of the gene are most prevalent, with, on average, 64% of isoniazid-resistant clinical isolates worldwide carrying a *katG* 315 mutation^3,4^. Moreover, one particular amino acid substitution (serine to threonine) accounts for 95% of all *katG* 315 mutations^4^. Mutations in *katG* are associated with a wide range of moderate- to high-level isoniazid resistance, above the commonly tested concentrations of 0.2 and 1 mg/L in solid- and 0.1 and 0.4 mg/L in liquid medium^5^. The *katG* Ser315Thr mutation in particular is associated with minimum inhibitory concentrations (MICs) ranging from 2 to >10 mg/L^5^, while (partial) deletion of the *katG* gene causes very high MICs (>25.6 mg/L)^6,7^.

In addition to *katG* mutations, isoniazid resistance arises from mutations in the promoter region of *inhA*, which lead to overexpression of isoniazid’s target InhA, requiring higher doses of the drug to achieve complete inhibition^3^. Mutations in the promoter region of *inhA* tend to result in low-level phenotypic resistance^5^ and also confer resistance to the second-line drugs ethionamide and prothionamide^3^. The most prevalent *inhA* promoter region mutation is the c-15t mutation, which is present in, on average, 19% of isoniazid-resistant clinical isolates worldwide^4^. In addition to the two most frequent causes of isoniazid resistance, mutations in *katG* expression regulatory genes (e.g. the *furA-katG* intergenic region and *sigI*) and in the coding region of *inhA*, as well as mutations involving isoniazid inactivation, redox potential alteration, mycothiol biosynthesis alteration and drug excretion by efflux pumps, have been described^3^.

Combinations of mutations in *katG* and the *inhA* promoter region confer high-level resistance (MICs > 10 mg/L)^8^. However, these variations do not explain the entire phenotypic heterogeneity observed for *katG* mutations. We therefore hypothesized that other (combinations of) genetic mutations contribute to the wide range of MICs observed among isoniazid-resistant isolates of *Mycobacterium tuberculosis*.

The therapeutic range of isoniazid is 3–6 mg/L^9^, typically achieved with a normal 300 mg dose for patients with a body weight >50 kg^10^, although serum levels can vary^11^ due to multiple factors, including a fatty meal, leading to lower peaks^9^, and the patient’s acetylator status^12^. Whereas treatment with isoniazid is often ceased when isoniazid resistance has been demonstrated at the 0.2 mg/L (solid medium) or 0.1 mg/L (liquid medium) cut-off, there is evidence that for low- to moderate-level resistance, isoniazid can have some remaining effectiveness at normal or elevated doses^5,13–18^. Recent WHO guidelines for the treatment of MDR-TB recommend the 9-month ‘Bangladesh regimen’, which includes a double dose of isoniazid (600 mg, rather than 300 mg)^19,20^.

Whereas conventional drug susceptibility testing takes days to weeks due to the slow growth rate of *M. tuberculosis*, novel molecular tests are able to analyse multiple genes simultaneously and provide the results within hours to days. The widely used commercial line probe assays (LPAs) for the detection of resistance to rifampicin and isoniazid are commonly thought to indicate high-level resistance to isoniazid if a *katG* mutation is detected, rendering the drug useless^21^. To shed some light on the highly variable MICs, even for the same *katG* mutation, we investigated which (combinations of) genetic mutations predict the level of phenotypic isoniazid resistance in clinical *M. tuberculosis* isolates. Incorporating this genetic information in rapid molecular tests would facilitate optimized use of isoniazid, one of the most potent anti-TB drugs, thereby enhancing effective and safe treatment of drug-resistant TB.

## Methods

### Ethics

Ethical approval for the Bangladesh treatment regimen and research methods was obtained from the Bangladesh Medical Research Council, Dhaka, Bangladesh^15^. Before enrolment, each patient completed and signed an informed consent form in the local language^15^. Ethical approval for this sub-study on the *M. tuberculosis* isolates was obtained from the ITM Institutional Review Board (IRB/AB/ac/125) and the analyses were performed in an ISO 15189 accredited environment.

### *M. tuberculosis* datasets

Two sets of *M. tuberculosis* isolates were selected for the present study (Supplementary Fig. S1). The first set includes 129 well-characterized strains from the WHO/TDR-TB Strain Bank (hereafter referred to as WHO-TDR strains) whose whole genomes had been sequenced on an Illumina HiSeq (ENA Short Read Archive accession PRJEB11653)^22^. The WHO/TDR-TB Strain Bank holds strains of diverse geographical origin with diverse resistance profiles to first- and second-line drugs (Supplementary Table S1)^23^. In addition to these 129 strains, four other WHO-TDR strains had whole genome sequences available yet were excluded, three due to heteroresistance as they were found to have a mutation in *katG* in 21%, 29% and 43% of reads (using PhyResSE, see below) and one because a mixture of resistant as well as susceptible bacilli for multiple drugs was suggestive of mixed infection. Isoniazid MIC determination on Löwenstein-Jensen (LJ) medium using concentrations of 0.05, 0.2, 0.8, 1.6 and 3.2 mg/L had been carried out previously for all 129 strains using the same method as described below^23^. These MIC values were included in this study. WHO-TDR strains having an isoniazid MIC ≥1.6 mg/L (n = 61) were selected for further isoniazid MIC determination in this study using higher drug concentrations.

The second set comprised isolates derived from baseline samples of MDR-TB patients from the Bangladesh cohort^15,16^. Patients were treated with a double dose isoniazid (7–12 mg/kg) as part of a 7-drug standardized MDR-TB regimen^15,16^. Only isolates that were highly resistant to fluoroquinolones (gatifloxacin MIC >1 mg/L on LJ) (n = 49) were included in order to enrich the panel with patients with an extensive TB treatment history resulting in highly isoniazid-resistant isolates. One isolate was excluded since it was no longer viable. Isoniazid MIC determination and whole genome sequencing (WGS) were carried out on the remaining 48 isolates. Subsequently, another isolate was excluded because it was found to have a mutation in the *inhA* promoter region in 48% of reads, suggesting isoniazid heteroresistance. As a result, 47 isolates were included in the analysis.

### Phenotypic drug susceptibility testing

Isoniazid MIC (MIC99, defined as the lowest drug concentration that inhibits 99% of visible bacillary growth) was determined for 61 WHO-TDR strains and 48 Bangladesh isolates on LJ medium using concentrations of 1.6, 3.2, 6.4, 12.8, 19.2 and 25.6 mg/L^24^. H37Rv (BCCM/ITM083715) and an MDR strain (BCCM/ITM021617) routinely used for quality control of in-house prepared media were run in tandem as a negative and positive control, respectively. MDR was defined as concurrent resistance to isoniazid and rifampicin, and non-MDR as resistance to isoniazid or rifampicin, but not both, or susceptibility to both. MICs were classified into four resistance groups, i.e. susceptible (MIC ≤ 0.2 mg/L), low-level resistant (0.8 ≤ MIC ≤ 1.6 mg/L, i.e. MICs larger than or equal to 0.8 mg/L but smaller than or equal to 1.6 mg/L), moderate-level resistant (3.2 ≤ MIC ≤ 12.8 mg/L, i.e. MICs larger than or equal to 3.2 mg/L but smaller than or equal to 12.8 mg/L) and high-level resistant (MIC ≥ 19.2 mg/L). Due to a lack of data in the literature on high isoniazid MIC frequencies and in particular their clinical relevance^5–8^, the border between moderate-level and high-level resistance was defined arbitrarily, based on the frequency distributions observed in this study. The groups were used in all subsequent analyses.

### gDNA extraction and whole genome sequencing

Whereas WGS data were already available for the WHO-TDR strains^22^, genomic DNA (gDNA) extraction and WGS were carried out for 48 Bangladesh isolates (NCBI Sequence Read Archive, BioProject accession PRJNA343956). To this end, colonies from LJ medium were heat-killed in 150 µL 0.5 M Tris-EDTA buffer. To each sample, 150 µL 30% sucrose solution, 50 µL 100 mg/mL lysozyme (Sigma-Aldrich, St. Louis, USA) and 20 µL 10 mg/mL RNase A (Life Technologies, Carlsbad, USA) were added, followed by shaking (200 rpm) at 37 °C for one hour. Subsequently, 100 µL 20% SDS, 40 µL 2.5 mg/mL proteinase K (MP Biomedicals, Santa Ana, USA) and approximately 50 µL glass beads (150–212 μm, Sigma-Aldrich, St. Louis, USA) were added to each sample, followed by shaking (200 rpm) at 37 °C overnight. Samples were then heated at 70 °C for 5 minutes and agitated using the FastPrep-24 (MP Biomedicals, Santa Ana, USA) at speed 5.0 for two times 25 seconds, followed by centrifugation. The Maxwell 16 Cell DNA Purification Kit (Promega, Madison, USA) was used to purify the extracted gDNA according to the manufacturer’s instructions. Fragmentation and purity were examined through gel electrophoresis and yield was measured using the Qubit dsDNA BR Assay Kit (Life Technologies, Carlsbad, USA) according to the manufacturer’s instructions. Extracted gDNA was sequenced (next generation sequencing) on an Illumina MiSeq platform using the Illumina Nextera XT DNA Library Preparation Kit.

### Genotypic-phenotypic correlation

To check for genetic mutations (primarily single-nucleotide polymorphisms (SNPs)) known to confer resistance to isoniazid, Illumina reads were uploaded to an online tool called Phylo-Resistance Search Engine (PhyResSE) between July and October 2015^25^. After the removal of low quality reads based upon their Quality score (Q < 20), PhyResSE maps the reads against the *M. tuberculosis* H37Rv reference genome (GenBank ID: NC_000962.3)^26,27^ and pre-processes the results. Subsequently, variants are determined and compared with a list of resistance-conferring and lineage-specific mutations^25^. This list is based on well-known mutations collected from the literature supplemented by the author’s in-house data and is regularly updated (during the study period no isoniazid resistance-conferring mutations were added)^25^. In addition, mutations for which strong experimental evidence exists are distinguished as high-confidence mutations^25^.

In order to search for all SNPs and insertions/deletions (indels) outside of well-known drug resistance-related positions of the genome, whole genome sequences were assembled from the reads of the final set of 129 WHO-TDR strains and 47 Bangladesh isolates with the Snippy software (version 3.0)^28^. Snippy compares reads to the *M. tuberculosis* H37Rv reference genome (NC_000962.3)^26,27^, employing the variant caller FreeBayes (version 0.9.21–7-g7dd41db)^29^. SNPs and indels found by Snippy were analysed in two ways. First, they were searched for low-confidence mutations. Included were all previously described isoniazid resistance-conferring mutations reviewed by Vilchèze and Jacobs^3^ and TBDReaMDB^30^, as well as any other non-synonymous mutations in the genes in which these previously described mutations were found. Second, to search for unknown (combinations of) resistance-conferring mutations, SNPs and indels were analysed using PICA (version 1.0.1) for each *M. tuberculosis* dataset separately^31^. PICA comprises a genotype-phenotype association rule method that searches for combinations of mutations that are characteristic of a certain phenotype (i.e. resistance level in this study)^31^. In order to search for (combinations of) genes associated with resistance, as opposed to individual mutations, PICA was also applied on SNPs and indels grouped by gene. Association rules found by PICA having a LaPlace association measure >0.95 were considered to be reliable.

Subsequently, a SNP alignment was made from the Snippy output for each dataset using an in-house Python script. Based on this SNP alignment, a maximum likelihood tree was built using Randomized Axelerated Maximum Likelihood (RAxML) (version 8.2.4)^32^ with Stamatakis ascertainment bias correction^33,34^ and a GTR + CAT model of evolution. The distribution of drug resistance-related mutations was correlated with the different phylogenetic lineages using the online interactive Tree Of Life (iTOL) tool^35^.

### Analysis

To test whether the presence of a (combination of) high-confidence mutation(s) was significantly associated with the isoniazid resistance level, Pearson’s chi-squared tests (α = 0.01 (rather than α = 0.05, to correct for multiple comparisons) were carried out for mutations found in more than two strains in the same dataset (IBM SPSS Statistics 23). For the WHO-TDR strains, the relative frequencies of these mutations found in each of the isoniazid-resistant groups (low-, moderate- and high-level) were compared to the relative frequencies in the susceptible group (groups as described below). For the Bangladesh isolates, the relative frequencies of the mutations found were compared between the moderate- and high-level resistant groups, since these isolates were all moderate- to high-level resistant. For low-confidence mutations, Pearson’s chi-squared tests (α = 0.01 (rather than α = 0.05, to correct for multiple comparisons) were carried out to test whether these mutations, grouped by gene, were significantly associated with the isoniazid resistance level. WHO-TDR and Bangladesh data were pooled.

### Data availability

Isoniazid MICs as well as high- and low-confidence mutations are available in this article and its Supplementary Information file. WGS reads are available in the ENA SRA (WHO-TDR strains, https://www.ebi.ac.uk/ena/data/view/PRJEB11653) and the NCBI SRA (Bangladesh isolates, https://www.ncbi.nlm.nih.gov/bioproject/PRJNA343956/). In addition, Bangladesh WGS reads and associated genotypic and phenotypic data have been submitted to the Relational Sequencing TB Data Platform (ReSeqTB) and will be publicly available upon publication of this article.

## Results

### Phenotypic resistance to isoniazid

**Figure 1:**
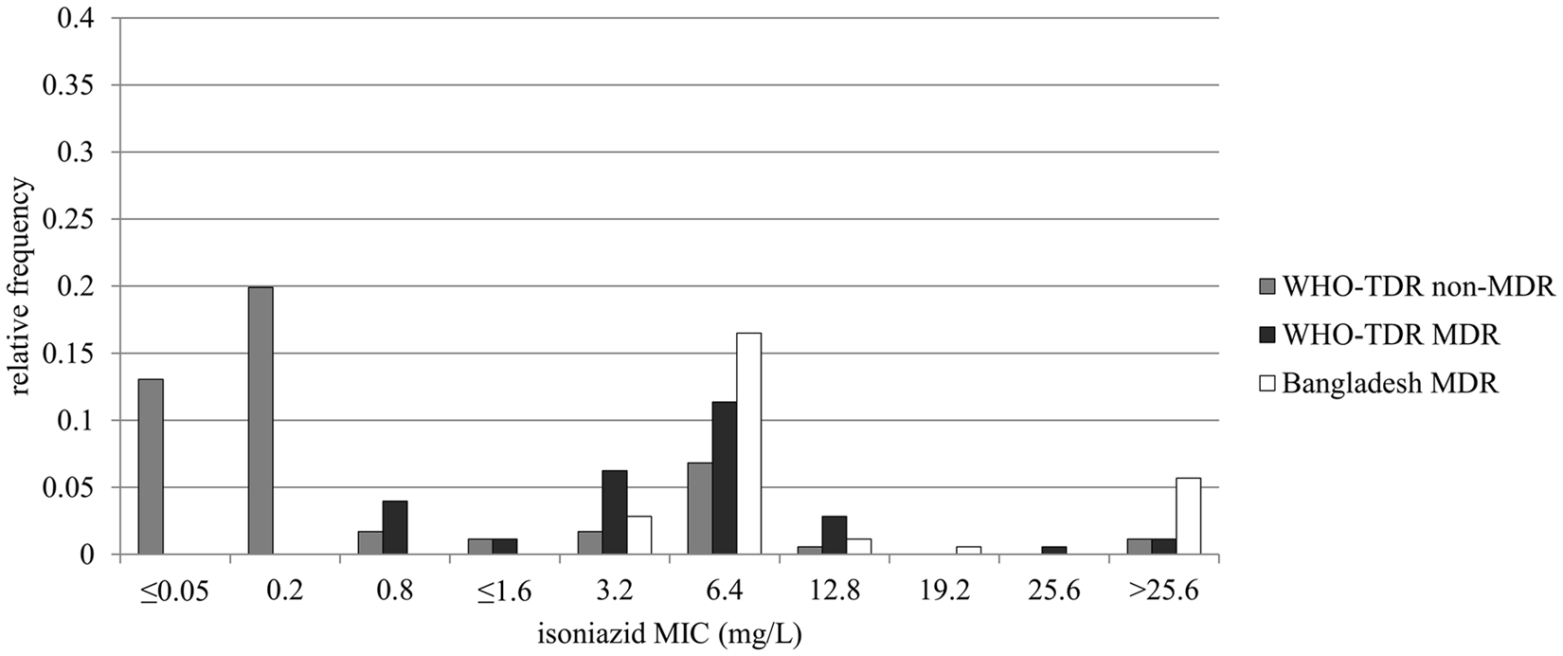
Relative frequency distributions of the isoniazid MICs of the WHO-TDR and Bangladesh isolates. MICs are depicted separately for MDR (i.e. concurrently resistant to isoniazid and rifampicin) and non-MDR isolates (i.e. resistant to isoniazid or rifampicin, but not both, or susceptible to both), given the assumption that cumulative drug exposure, including to isoniazid, would have been higher for most MDR patients. WHO-TDR strains with an MIC up to and including 0.8 mg/L had this MIC determined previously. All other MICs were determined in this study, i.e. 1.6 mg/L was the lowest concentration used in this study. MDR = multidrug-resistant; MIC = minimum inhibitory concentration.

Isoniazid-resistant isolates (MIC > 0.2 mg/L, based on the proportion method cut-off for isoniazid^5^) were characterized by a mode of 6.4 mg/L and a second, smaller peak representing MICs ≥25.6 mg/L (Fig. 1). The Bangladesh MDR isolates (n = 47) tended to have a higher isoniazid MIC than the WHO-TDR MDR strains (n = 48), although their mode and median were the same (both 6.4 mg/L) (Bangladesh MDR versus WHO-TDR MDR, Fig. 1). The WHO-TDR collection included 58 isoniazid-susceptible strains.

### Association between high-confidence mutations and phenotypic resistance level

**Figure 2:**
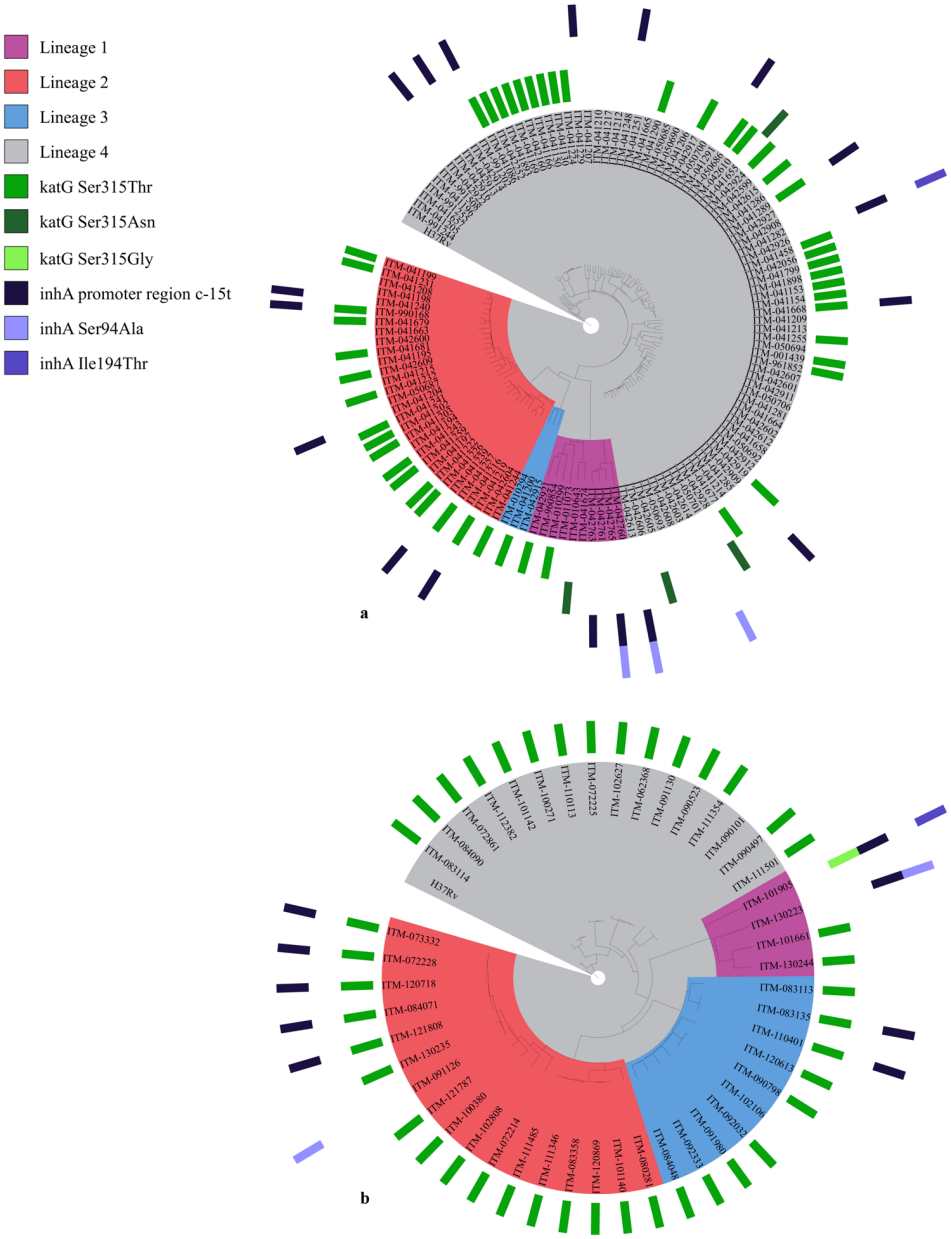
Maximum likelihood trees of the WHO-TDR (**a**) and Bangladesh (**b**) isolates (rooted at the H37Rv reference genome). For each isolate, high-confidence mutations and lineage are depicted at the outside of the tree and along the corresponding branch, respectively.

**Figure 3:**
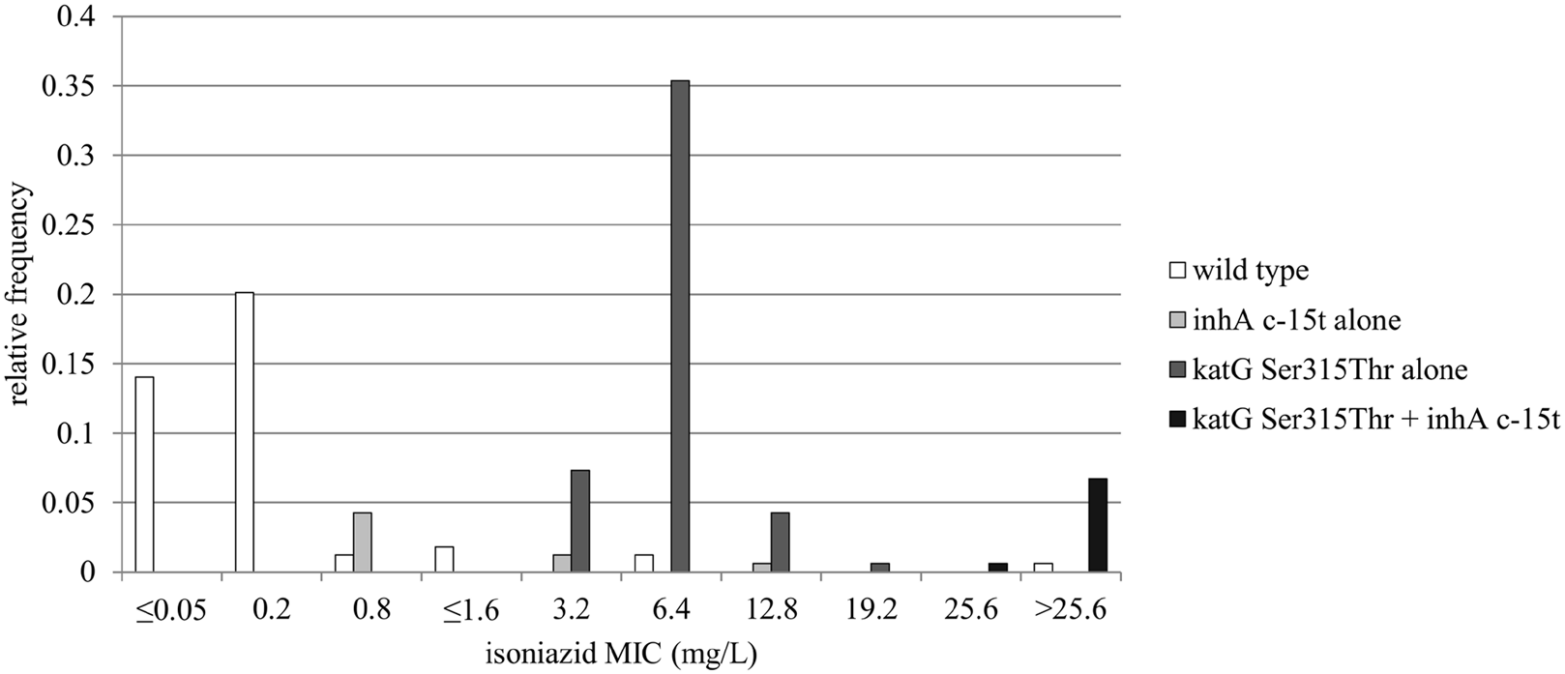
Isoniazid MIC relative frequency distributions of isolates harbouring the *inhA* promoter region c-15t mutation alone, the *katG* Ser315Thr mutation alone, the combination of these two mutations, or no high-confidence mutation for isoniazid (wild type). WHO-TDR and Bangladesh isolates were pooled to allow comparison of low frequencies. WHO-TDR strains with an MIC up to and including 0.8 mg/L had this MIC determined previously. All other MICs were determined in this study, i.e. 1.6 mg/L was the lowest concentration used in this study. MIC = minimum inhibitory concentration.

High-confidence mutations, alone or in combination, were detected in 65/71 phenotypically isoniazid-resistant WHO-TDR strains and 45/47 Bangladesh isolates, while 1 of the 58 susceptible isolates had a mutation in *inhA* (Table 1 and Supplementary Tables S1 and S2). Among these high-confidence mutations, the *katG* Ser315Thr mutation alone (for WHO-TDR in 43/66 and for Bangladesh in 35/45 isolates with any high-confidence mutation), the *inhA* promoter region c-15t mutation alone (WHO-TDR 10/66) and the combination of both (WHO-TDR 5/66 and Bangladesh 7/45) were observed most frequently. For both the WHO-TDR strains (Fig. 2a) and the Bangladesh isolates (Fig. 2b), the high-confidence mutations were widely spread across the different phylogenetic lineages, consistent with the known convergent evolution of isoniazid resistance mutations^36^. The *inhA* promoter region c-15t mutation alone was present significantly more often in the low-level resistant group compared to the susceptible group, but not in the moderate- and high-level resistant groups (WHO-TDR, *p* = 0.001). Similarly, the *katG* Ser315Thr mutation alone was present significantly more often in the moderate-level resistant group compared to the susceptible group (WHO-TDR, *p* = 0.001) or the high-level resistant group (Bangladesh, *p* = 0.001). Lastly, the combination of the *katG* Ser315Thr mutation with the c-15t mutation in the *inhA* promoter region was present exclusively and significantly more often in the high-level resistant group compared to the susceptible group (WHO-TDR, *p* = 0.001) or the moderate-level resistant group (Bangladesh, *p* = 0.001). Isoniazid MIC relative frequency distributions of isolates harbouring the *inhA* promoter region c-15t mutation alone, the *katG* Ser315Thr mutation alone, the combination of these two mutations, or no high-confidence mutation for isoniazid (wild type), are shown in Fig. 3.

**Table 1:** (Combinations of) high-confidence mutations detected in the WHO-TDR (a) and Bangladesh (b) isolates.

	susceptible		low-level resistance		moderate-level resistance		high-level resistance	
	n	%	n	%	n	%	n	%
**a** WHO-TDR								
wild type	57	98.3%	5	35.7%	1	1.9%	0	0.0%
*katG* Ser315Thr alone	0	0.0%	0	0.0%	43	82.7% (***p* =0. 000**)	0	0.0%
*katG* Ser315Asn alone	0	0.0%	2	14.3% (***p* =** 0.**004**)	2	3.8%	0	0.0%
*inhA* promoter region c-15t alone	0	0.0%	7	50.0% (***p* =** 0.**000**)	3	5.8%	0	0.0%
*inhA* Ser94Ala alone	1	1.7%	0	0.0%	0	0.0%	0	0.0%
*katG* Ser315Thr + *inhA* promoter region c-15t	0	0.0%	0	0.0%	0	0.0%	5	100% (***p* =** 0.**000**)
*inhA* promoter region c-15t + *inhA* Ser94Ala	0	0.0%	0	0.0%	2	3.8%	0	0.0%
*inhA* promoter region c-15t + *inhA* Ile194Thr	0	0.0%	0	0.0%	1	1.9%	0	0.0%
total	58	100%	14	100%	52	100%	5	100%
**b** Bangladesh								
wild type					1	2.8%	1	9.1%
*katG* Ser315Thr alone					34	94.4% (***p* =** 0.**000**)	1	9.1%
*inhA* Ser94Ala alone					1	2.8%	0	0.0%
*katG* Ser315Thr + *inhA* promoter region c-15t					0	0.0%	7	63.6% (***p* =** 0.**000**)
*katG* Ser315Gly + *inhA* promoter region c-15t + *inhA* Ile194Thr					0	0.0%	1	9.1%
*inhA* promoter region c-15t + *inhA* Ser94Ala					0	0.0%	1	9.1%
Total					36	100%	11	100%

susceptible: MIC ≤ 0.2 mg/L; low-level resistance: 0.8 ≤ MIC ≤ 1.6 mg/L; moderate-level resistance: 3.2 ≤ MIC ≤ 12.8 mg/L; high-level resistance: MIC ≥ 19.2 mg/L.

### Low-confidence mutations and mutations detected using association rule mining

In addition to the detection of high-confidence mutations, whole genome sequences were used to search for low-confidence mutations as well as for unknown (combinations of) SNPs and indels or (combinations of) genes that were present/mutated more often in one of the resistant groups compared to the other groups. Previously described low-confidence mutations were found in 58/129 WHO-TDR strains and 37/47 Bangladesh isolates (Supplementary Tables S1, S2 and S3). Newly found non-synonymous mutations in genes in which mutations were described previously, were found in 95/129 WHO-TDR strains and 39/47 Bangladesh isolates (Supplementary Tables S1, S2 and S3). However, no mutations were associated with a particular MIC or resistance level or explained the variation in isoniazid MICs of isolates with a *katG* Ser315Thr mutation. Grouping previously described and newly found low-confidence mutations by gene showed that mutations in *Rv0340* were significantly associated with high-level resistance (*p* = 0.008) and close to significantly associated with moderate-level resistance (*p* = 0.020) (Table 2). However, all phenotypically resistant isolates with a mutation in *Rv0340* (n = 14) had high-confidence mutations as well, and all but one had an isoniazid MIC representative of the resistance level associated with these high-confidence mutations, which confounds the associations between *Rv0340* mutations and resistance level. Previously described and newly found low-confidence mutations in *katG* were significantly associated with low-level isoniazid resistance (*p* = 0.000) (*katG* Arg463Leu was excluded from this calculation since it has been reported as not associated with isoniazid resistance)^37,38^ (Table 2). Among isolates without high-confidence mutations, previously described low-confidence mutations were found in 1/6 WHO-TDR strains (*oxyR’-ahpC* intergenic region c-52t) and 2/2 Bangladesh isolates (*katG* Arg463Leu, *fbpC* Gly158Ser, *Rv2242* Met323Thr, *inhA* promoter region g-17t, and *oxyR’-ahpC* intergenic region g-88a in one isolate and *oxyR’-ahpC* intergenic region c-81t in the other isolate). However, these mutations were found in susceptible isolates as well (29.3% *katG* Arg463Leu, 3.4% *fbpC* Gly158Ser, and 3.4% *Rv2242* Met323Thr) or occurred in only one isolate without high-confidence mutations (*inhA* promoter region g-17t, *oxyR’-ahpC* intergenic region c-52t, *oxyR’-ahpC* intergenic region c-81t, and *oxyR’-ahpC* intergenic region g-88a) (Supplementary Tables S1 and S2). Newly found low-confidence mutations in *katG* were found in 4/6 WHO-TDR strains without high-confidence mutations (all four were low-level resistant), along with other previously not described low-confidence mutations (Supplementary Tables S1). No unknown (combinations of) SNPs and indels (either grouped by gene or not) searched for with association rule mining were found to be reliably associated with one resistance group compared to the other groups.

**Table 2:** Frequency of low-confidence mutations, both previously described low-confidence mutations^3,30^ and newly found mutations in genes in which mutations were described previously.

	susceptible (n = 58)		low-level resistance (n = 14)		moderate-level resistance (n = 88)		high-level resistance (n = 16)	
	n	%	n	%	n	%	n	%
*accD6*	13	22.4%	4	28.6%	30	34.1%	7	43.8%
*ahpC*	0	0%	0	0%	1	1.1%	0	0%
*efpA*	1	1.7%	1	7.1%	1	1.1%	0	0%
*fabD*	2	3.4%	0	0%	4	4.5%	1	6.3%
*fabG1*	0	0%	0	0%	0	0%	1	6.3%
*fadE24*	3	5.2%	1	7.1%	13	14.8%	0	0%
*fbpC*	2	3.4%	0	0%	9	10.2%	2	12.5%
*furA*	1	1.7%	0	0%	0	0%	0	0%
inhA*	0	0%	0	0%	1	1.1%	0	0%
*inhA* promoter region*	0	0%	0	0%	4	4.5%	0	0%
*iniA*	7	12.1%	3	21.4%	13	14.8%	5	31.3%
*iniB*	1	1.7%	0	0%	3	3.4%	0	0%
*iniC*	1	1.7%	1	7.1%	2	2.3%	0	0%
*kasA*	2	3.4%	1	7.1%	6	6.8%	0	0%
katG*	2	3.4%	5	35.7% (***p* =** 0.**000**)	9	10.2%	2	12.5%
*katG* Arg463Leu**	17	29.3%	4	28.6%	44	50.0%	11	68.8%
*nat*	1	1.7%	0	0%	5	5.7%	0	0%
*ndh*	1	1.7%	0	0%	1	1.1%	1	6.3%
*oxyR’-ahpC* intergenic region	3	5.2%	1	7.1%	11	12.5%	3	18.8%
*Rv0340*	1	1.7%	0	0%	11	12.5%	3	18.8%
*Rv1592c*	38	65.5%	8	57.1%	50	56.8%	13	81.3%
*Rv1772*	0	0%	0	0%	3	3.4%	0	0%
*Rv2242*	3	5.2%	0	0%	9	10.2%	2	12.5%

Mutations were grouped by gene and WHO-TDR and Bangladesh isolates were pooled. Percentages given are based on the total number of isolates in each resistance group. *In this table only low-confidence mutations were included; **katG Arg463Leu has been reported as not associated with isoniazid resistance and is therefore shown in a separate row^37,38^; susceptible: MIC ≤ 0.2 mg/L; low-level resistance: 0.8 ≤ MIC ≤ 1.6 mg/L; moderate-level resistance: 3.2 ≤ MIC ≤ 12.8 mg/L; high-level resistance: MIC ≥ 19.2 mg/L.

## Discussion

This study, through the systematic linking of extensive isoniazid MIC data with genomic mutations, suggests that the combination of well-characterized, easily detectable mutations can reliably predict excessively high resistance of *M. tuberculosis* to isoniazid. We found that the combination of *inhA* promoter region and *katG* mutations is associated with the highest-level resistance (≥19.2 mg/L), exceeding peak level concentrations of the drug even at the highest doses in clinical use. Our results are largely in agreement with the associations between known resistance-conferring mutations and the level of phenotypic resistance described in the literature, typically tested at lower concentrations^5–8^. However, exceptions were not rare for single *inhA* promoter mutations, with sometimes not a low- but moderate-level MIC, similar to *katG* mutations. However, the mode of MICs associated with *katG* was at the upper limit of the peak isoniazid serum concentration at normal dosing (3–6 mg/L). In addition, the specificity of high-confidence mutations is high, as previously shown^4^.

Although previous research found that certain low-confidence mutations, e.g. in the *oxyR’*-*ahpC* intergenic region, contribute to the detection of phenotypically isoniazid-resistant isolates^4^, in this study, except for the association between low-confidence mutations in *katG* and low-level resistance, no overt association between low-confidence or unknown mutations and isoniazid resistance (level) was found. Although confounded by the association with high-confidence mutations, the association between mutations in *Rv0340* and isoniazid resistance found in this study calls for further investigation, especially given the fact that *Rv0340* is located adjacent to the *iniBAC* operon, which has previously been found to play a potential role in isoniazid resistance^3^.

The analysis of low-confidence mutations was hampered by the relatively small sample size of our study, resulting in low frequencies of these mutations. Moreover, the selection criteria for the WHO-TDR and Bangladesh isolates have likely led to an overrepresentation of well-known resistance mutations and high levels of isoniazid resistance, respectively, which may limit the generalizability of our conclusion that a limited number of high-confidence mutations is able to predict the resistance level of the majority of isoniazid-resistant isolates.

Mutations in the promoter region of *inhA* were found by a previous study to not increase the risk of an unfavourable treatment outcome (treatment failure or death) upon first-line treatment with 2 months of isoniazid, rifampicin, streptomycin and pyrazinamide, followed by 6 months of isoniazid and ethambutol, although such mutations did increase the risk of relapse^13^. In contrast, *katG* 315 mutations were associated with an unfavourable treatment outcome^13^. Whether *katG* mutations still predict an unfavourable treatment outcome upon treatment with higher doses of isoniazid, remains to be resolved. Since the majority of *katG* Ser315Thr MICs remains below the serum peak of around 15 mg/L upon the WHO recommended dose of 15–20 mg/kg^19,39^, isoniazid might still have a more or less important remaining activity when used in an otherwise effective regimen^18,40^. The high level of phenotypic resistance (≥19.2 mg/L) associated with the combination of *katG* Ser315Thr and the c-15t mutation in the *inhA* promoter region however exceeds this serum peak, making it highly unlikely that even high-dose isoniazid would still yield some clinical activity.

Dose-related side effects of isoniazid are a concern in patients taking higher doses. A study by Fox *et al*.^40^ discussing pharmacodynamics in patients in Madras, India, states that for isoniazid doses from 2.2 to 13.9 mg/kg, the AUC was most predictive of the incidence of peripheral neuropathy, while treatment outcome was best explained by peak serum concentrations and isoniazid resistance. In the Bangladesh cohort, neuropathy was infrequently seen in patients on double-dose isoniazid and was manageable by concurrent administration of pyridoxine. A randomised controlled trial by Katiyar *et al*.^14^ comparing high-dose isoniazid (16–18 mg/kg) to normal dose (5 mg/kg) or placebo found a significantly higher incidence of peripheral neuropathy in the group that received high-dose isoniazid compared to the other groups, but no higher incidence of hepatotoxicity.

The strong association between the combination of *inhA* c-15t and *katG* Ser315Thr and high-level resistance could be of importance for the interpretation of current and future molecular diagnostic tests, since rapid prediction of the isoniazid resistance level is paramount for deciding whether to continue treatment with (high-dose) isoniazid or not. First-line LPAs like the Hain MTBDR*plus* can reliably identify isolates with these mutations and thus predict the isoniazid resistance level too high to overcome, because of the combined *katG* and *inhA* promoter mutations found in our study, or reportedly also due to a *katG* deletion^6,7^. But contrary to widespread belief, an isolated *katG* or *inhA* mutation can indicate highly variable MIC levels. *inhA* mutations do not always indicate very low and *katG* mutations not necessarily very high resistance levels, so that first-line LPA cannot be used to decide with high confidence that isoniazid will or will not be useful in a combination regimen when only one of both is detected. Moreover, further research is required to investigate whether high-dose isoniazid indeed contributes to successful outcome in patients with low- or moderate-level resistance, caused by a *katG* or *inhA* mutation alone.

## Electronic supplementary material

Dataset 1
